# DoGNet: A deep architecture for synapse detection in multiplexed fluorescence images

**DOI:** 10.1371/journal.pcbi.1007012

**Published:** 2019-05-13

**Authors:** Victor Kulikov, Syuan-Ming Guo, Matthew Stone, Allen Goodman, Anne Carpenter, Mark Bathe, Victor Lempitsky

**Affiliations:** 1 CDISE, Skoltech, Moscow, Russian Federation; 2 Department of Biological Engineering, Massachusetts Institute of Technology, Cambridge, Massachusetts, United States of America; 3 Imaging Platform, Broad Institute of Harvard and MIT, Cambridge, Massachusetts, United States of America; Johns Hopkins University, UNITED STATES

## Abstract

Neuronal synapses transmit electrochemical signals between cells through the coordinated action of presynaptic vesicles, ion channels, scaffolding and adapter proteins, and membrane receptors. In situ structural characterization of numerous synaptic proteins simultaneously through multiplexed imaging facilitates a bottom-up approach to synapse classification and phenotypic description. Objective automation of efficient and reliable synapse detection within these datasets is essential for the high-throughput investigation of synaptic features. Convolutional neural networks can solve this generalized problem of synapse detection, however, these architectures require large numbers of training examples to optimize their thousands of parameters. We propose DoGNet, a neural network architecture that closes the gap between classical computer vision blob detectors, such as Difference of Gaussians (DoG) filters, and modern convolutional networks. DoGNet is optimized to analyze highly multiplexed microscopy data. Its small number of training parameters allows DoGNet to be trained with few examples, which facilitates its application to new datasets without overfitting. We evaluate the method on multiplexed fluorescence imaging data from both primary mouse neuronal cultures and mouse cortex tissue slices. We show that DoGNet outperforms convolutional networks with a low-to-moderate number of training examples, and DoGNet is efficiently transferred between datasets collected from separate research groups. DoGNet synapse localizations can then be used to guide the segmentation of individual synaptic protein locations and spatial extents, revealing their spatial organization and relative abundances within individual synapses. The source code is publicly available: https://github.com/kulikovv/dognet.

## Author summary

Multiplexed fluorescence imaging of synaptic proteins facilitates high throughput investigations in neuroscience and drug discovery. Currently, there are several approaches to synapse detection using computational image processing. Unsupervised techniques rely on the *a priori* knowledge of synapse properties, such as size, intensity, and co-localization of synapse markers in each channel. For each experimental replicate, these parameters are typically tuned manually in order to obtain appropriate results. In contrast, supervised methods like modern convolutional networks require massive amounts of manually labeled data, and are sensitive to signal/noise ratios. As an alternative, here we propose DoGNet, a neural architecture that closes the gap between classical computer vision blob detectors, such as Difference of Gaussians (DoG) filters, and modern convolutional networks. This approach leverages the strengths of each approach, including automatic tuning of detection parameters, prior knowledge of the synaptic signal shape, and requiring only several training examples. Overall, DoGNet is a new tool for blob detection from multiplexed fluorescence images consisting of several up to dozens of fluorescence channels that requires minimal supervision due to its few input parameters. It offers the ability to capture complex dependencies between synaptic signals in distinct imaging planes, acting as a trainable frequency filter.

## Introduction

Neuronal synapses are the fundamental sites of electrochemical signal transmission within the brain that underlie learning and memory. The protein compositions within both presynaptic and postsynaptic synaptic densities crucially determine the stability and transmission sensitivity of individual synapses [1, 2]. The analysis of synapse protein abundances, localizations, and morphologies offers better understanding of neuronal function, as well as ultimately psychiatric and neurological diseases [3, 4]. However, the high spatial density and structural complexity of synapses both *in vitro* and *in vivo* requires new computational tools for the objective and efficient identification and structural profiling of diverse populations of synapses.

Fluorescence microscopy (FM) combines molecular discrimination with high-throughput, low-cost image acquisition of large fields of view of neuronal synapses within intact specimens using modern confocal imaging instruments. Immunostaining techniques [5, 6] can be used to identify synapses as puncta within fluorescence microscopy images to distinguish distinct types of synapses based on molecular composition. However, phenotypic classification of individual synapses in FM images is challenging because of the morphological complexities of variable structural features of synapses, including synaptic boutons, presynaptic vesicles, and synaptic clefts, which cannot be resolved using conventional light microscopy.

Manual synapse detection and classification quickly becomes intractable for even moderately sized datasets, thus necessitating automated processing. In recent years, deep convolutional neural networks (ConvNets) have become state-of-the-art tools for image classification [7] and segmentation [8], and have been extended to electron microscopy images of neuronal synapses [9, 10]. ConvNets, however, requires thousands of learnable parameters and therefore requires a large amount of training data to avoid overfitting. Furthermore, even when sufficient training data is available, ConvNets may fail to generalize to new experimental conditions that result in modified image properties. Both of these factors complicate the use of ConvNets for synapse detection in fluorescence microscopy images, often rendering traditional blob detection techniques such as [11] preferable.

In this work, we introduce a new neural network architecture for synapse detection in multiplexed immunofluorescence images. Compared with ConvNets, the new architecture achieves a considerable reduction in the number of learnable parameters by replacing the generic filters of ConvNets with Difference of Gaussians (DoG) filters [12].

This replacement is motivated by the fact that in FM images, typical mammalian synapses are close in size to the diffraction limit of light. Consequently, individual synapses are resolved as blobs due to the convolution of the microscope point spread function with the underlying fluorescence labels, and approximately Gaussian [13, 14]. DoG filters are known to be good blob detectors and have few parameters. The DoGNet architecture uses multiple trainable DoG filters applied to multiple input channels and, potentially, in a hierarchical way (Deep DoGNets). The parameters of the DoG filters inside DoGNets are trained in an end-to-end fashion together with other network layers. We use linear weights layer to combine the response maps of different DoG filters together into a probabilistic map.

**Fig 1:**
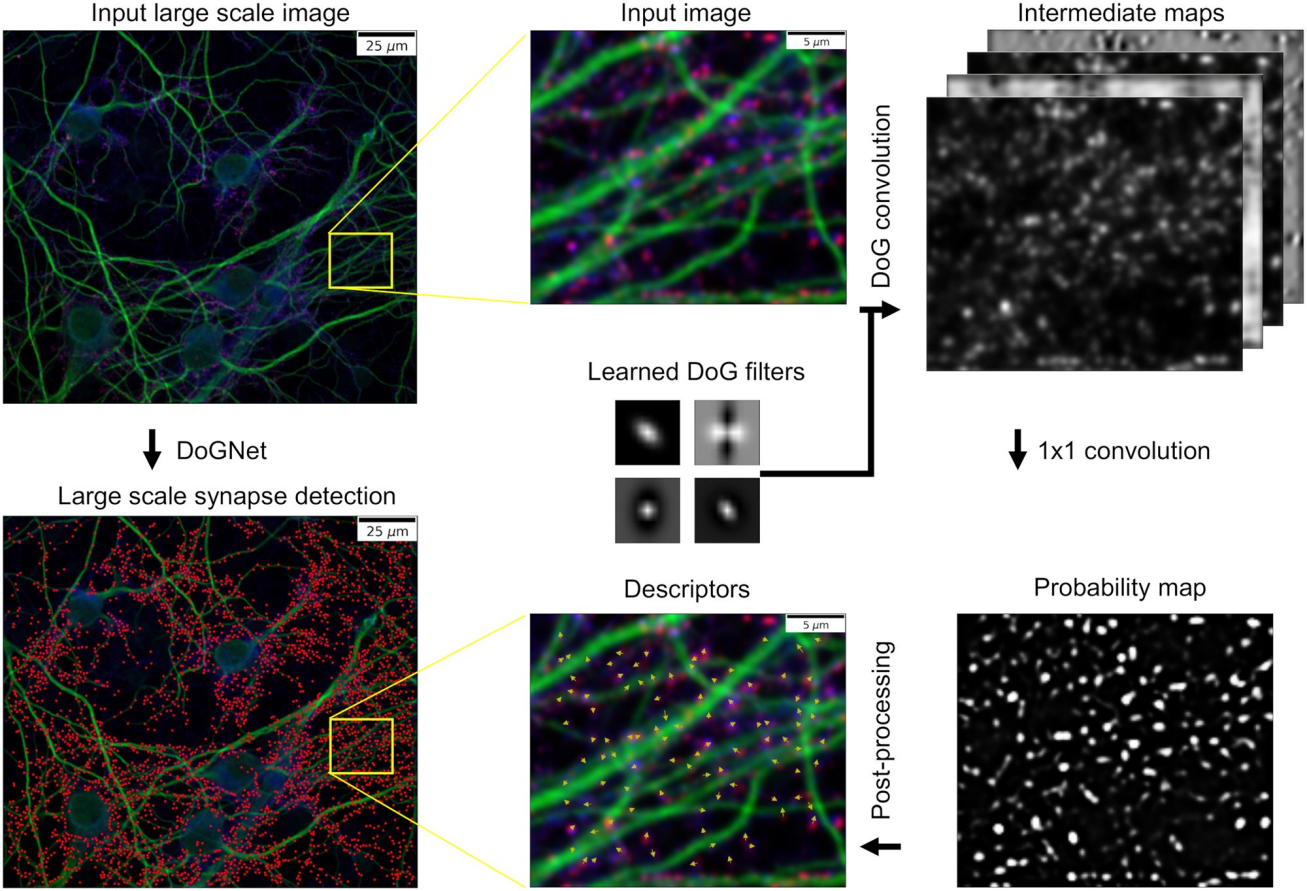
Single layer DoGNet inference pipeline. Synaptic protein channels from the PRISM [6] dataset are used as input images. Each channel of the input images are convolved with a number of the Difference of Gaussian filters. This processing is performed using the sigmoid function convolved with (or multipled by) the per-pixel weighted sum of intermediate maps. The DoGNet is trained to predict the probability map for each pixel as belonging to a synapse. Synapses locations and parameters of their proteins (such as average intensities and shapes) are extracted by fitting Gaussians to the intensities of individual proteins in the vicinities of the local maxima of the resulting probability map. The scalebar on the large scale image equals 25 *μ*m (5 *μ*m in the cropped region).

We post-process this probability map in order to estimate the centers of synapses and describe their properties. For each synapse, the output of our system gives the location and the shape of the punctum for each protein marker, with desired confidence level. The complete image processing pipeline is shown Fig 1.

We have validated the performance of this new architecture by comparing several variations of DoGNets to popular types of ConvNet architectures including U-Nets [8] and Fully Convolutional Networks [15] for the task of synapse detection. The comparison is performed on four different datasets including a synthetic dataset, an annotated real dataset from previous work [16, 17], and another human annotated dataset acquired with PRISM multiplexed imaging [6]. Apart from outperforming ConvNet architectures, the DoGNet approach achieves accuracy comparable to inter-human agreement on the dataset from [6]. Finally, we have shown that a DoGNet trained on one correlated Array Tomography and Electron Microscopy dataset can be successfully applied to an Array Tomography (AT) dataset without associated Electron Microscopy images, which may facilitate accurate synapse detection in large datasets where correlated EM data are not available.

Overall, the system is based on the DoGNet detector and a post-processing pipeline that reveals synaptic structure consistent with known synaptic protein localization, and provides a wealth of data for further downstream phenotypic analysis, thereby achieving successful automation of synapse detection in neuronal FM images. Notably, the DoGNet architecture is not specific to such images, and can be applied to other microscopy modalities where objects of interest show a punctate spatial patterning, or where, more generally, a certain image analysis task may be performed via learnable blob detection such as single molecule segmentation in super-resolution microscopy and single particle tracking [18], detection of clusters or endosomes in immunofluorescence images [19], and detection of puncta in fluorescence in situ hybridization (FISH) datasets [20, 21].

### Related work

Automation of synapse detection and large-scale investigation of neuronal organization has seen considerable progress in recent years. Most work has been dedicated to the segmentation of electron microscopy datasets, with modern high-throughput pipelines for automated segmentation and morphological reconstruction of synapses [8–10, 22, 23]. Much of this progress may be credited to deep convolutional networks. Segmentation accuracy of these approaches can be increased by making deeper networks [24], adding dilated/ a-trous convolution [25] or using hourglass architectures [8, 26] that include downscaling/upscaling parts with so-called *skip connections*. ConvNets typically outperform random forest and other classical machine learning approaches that are dependent on hand-crafted features such as those proposed in [27, 28]. At the same time, while it is possible to reduce the number of training examples needed by splitting the segmentation pipeline into several smaller pipelines [10], the challenge of reducnig the number of training parameters without sacrificing segmentation accuracy remains.

Within the context of neuronal immunofluorescence images, synapses are typically defined by the colocalization of pre- and postsynaptic proteins within puncta that have sizes on the order of the diffraction limit of 250 nm. One fully automated method using priors, which quantifies synaptic elements and complete synapses based on pre- and postsynaptic labeling plus a dendritic or cell surface marker, was previously proposed and applied successfully [29]. Alternatively, a machine learning approach to synapse detection was proposed in [30, 31], where a support vector machine (SVM) was used to estimate the confidence of a pixel being a synapse, depending on a small number of neighboring pixels. Synapse positions were then computed from these confidence values by evaluating local confidence profiles and comparing them with a minimum confidence value. Finally, in [32], a probabilistic approach to synapse detection on AT volumes was proposed. The principal idea of this approach was to estimate the probability of a pixel being a punctum within each tissue slice, and then calculating the joint distribution of presynapic and postsynapic proteins between neighbouring slices. Our work was mainly inspired by works [32] and [11], that produced the state-of-the-art results in synapse detection on fluorescence images.

More conventional machine vision techniques have also been applied for synapse detection [6, 11, 12]. These methods aim at detecting regions that differ in brightness compared with neighboring regions. The most common approach for this task is convolution with a Laplacian filter [12]. The Laplacian filter can be computed as the limiting case of the difference between two Gaussian smoothed images. Since convolution with a Gaussian kernel is a linear operation, convolution with the difference of two Gaussian kernels can be used instead of seeking the difference between smooth images. The usage of Difference of Gaussians for synapse detection was proposed in [11] with manually defined filter parameters. Here, we introduce a new DoGNet architecture that integrates the use of simple DoG filters for blob detection with machine, deep learning, thereby combining the strengths of the preceding published approaches [8, 11, 32]. Our approach offers the ability to capture complex dependencies between synaptic signals in distinct imaging planes, acting as a trainable frequency filter.

## Materials and methods

**Fig 2:**
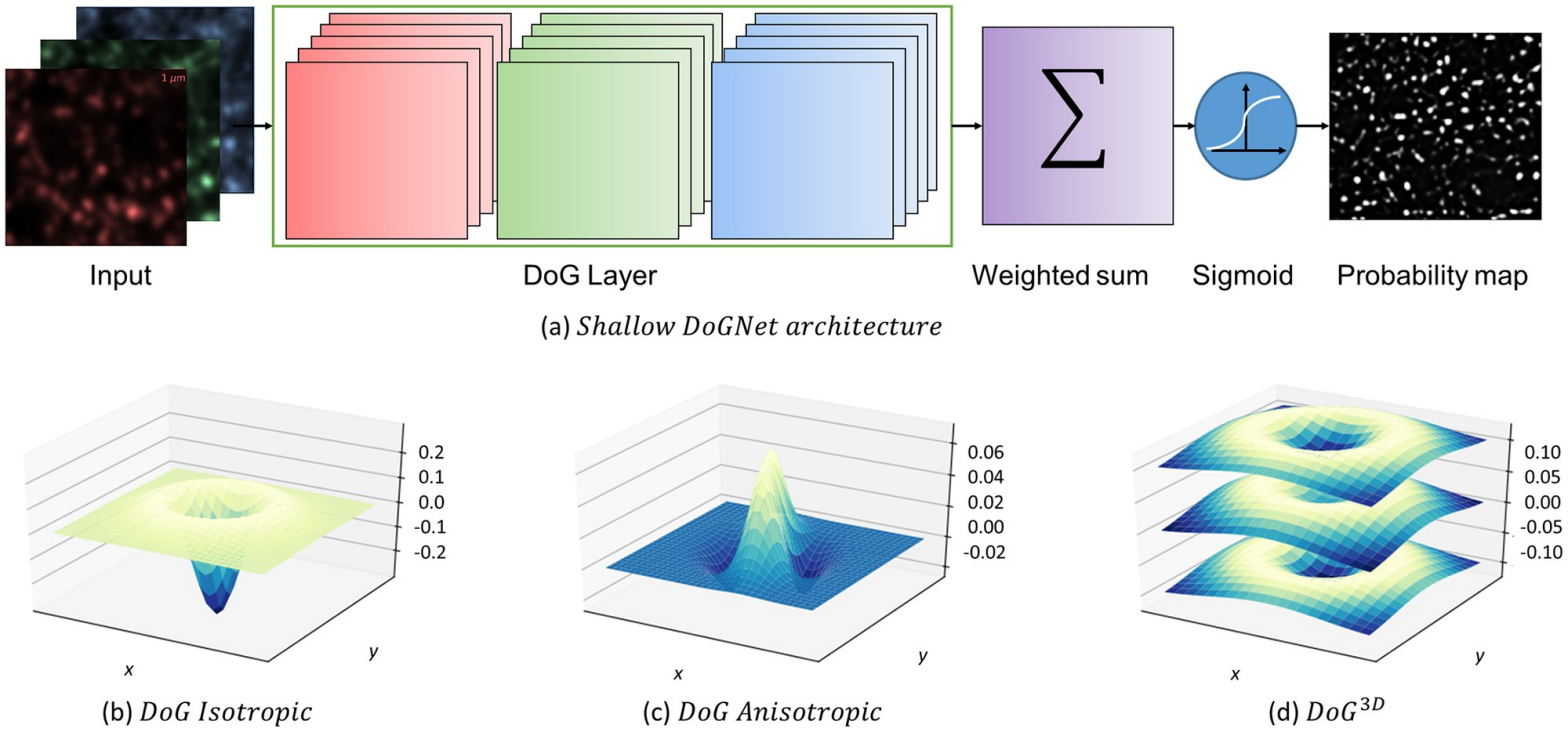
(a) The architecture of shallow DoGNet. The input image channels (for example synapsin, vGlut, and PSD95) are each processed by five trainable DoG filters. The weighted sum (with trainable weights) combines the resulting 15 DoG layer output maps into a single map. The sigmoid function converts the latter map into a pixel probability map. (b,c,d) The variations of the Difference of Gaussians that we use in each DoG layer. (b) An isotropic Difference of Gaussians. (c) An anisotropic difference of Gaussians. Each Gaussian is described by a pair of variance values and a rotation angle. (d) A 3D Isotropic Difference of Gaussians. Surfaces show filter values along *z* slices.

Our synapse puncta detection procedure consists of two steps: an application of the pre-trained DoGNet architecture to imaging planes of the source image and a post-processing of its output. In a nutshell, DoGNet is a standard convolutional neural network with convolution kernels reparametrized using the Difference-of-Gaussians (DoG) as shown in Fig 2. The DoGNet architecture applies a small number of DoG filters to each protein channel and then combines the outputs of the filtering operations. We train that network end-to-end using the backpropagation algorithm [33]. Accordingly, we describe the operation of our procedure by first discussing the properties of trainable DoG filters. We then discuss single layer and deep versions of the DoGNet architecture, and the training processes for both. Finally, we present in detail the post-processing procedure.

### Difference-of-Gaussians filters

In classical computer vision, the DoG filter is perhaps the most popular operation for blob detection. As follows from its name, DoG filtering corresponds to applying two Gaussian filters to the same real-valued image and then subtracting the results. As the difference between two different low-pass filtered images, the DoG is actually a band-pass filter, which removes high frequency components representing noise as well as some low frequency components representing the background variation of the image. The frequency components in the preserved band are assumed to be associated with the edges and blobs that are of interest. DoG filters are often regarded as approximations to Laplacian-of-Gaussian filters that require more operations to compute.

Depending on the parameterization of the underlying Gaussian filters, DoG filters may vary in their complexity. For example, in the most common case, one considers the difference of two isotropic Gaussian probability distribution functions as the filter kernel:

$$\begin{array}{c} \text{DoG}\;\text{Isotropic}\left[w_{1},w_{2},\sigma_{1},\sigma_{2}\right]\left(x,y\right)=w_{1}\text{exp}(-\frac{x^{2}+y^{2}}{2\sigma_{1}^{2}})-w_{2}\text{exp}(-\frac{x^{2}+y^{2}}{2\sigma_{2}^{2}}) \end{array}$$ (1)

This version of the DoG filter depends on four parameters, namely the amplitude coefficients *w*_1_ and *w*_2_, as well as the bandwidth parameters *σ*_1_ and *σ*_2_. The shape of the resulting function is depicted in Fig 2(b). The amplitudes *w*_1_ and *w*_2_ can be replaced by normalizing coefficients 1/2*πσ*_1_ and 1/2*πσ*_2_ respectively, reducing the number of trainable parameters to just two.

The four- and the two-parameter DoG filters described above are suitable for detecting isotropic blobs. For anisotropic blob detection, pairs of anisotropic Gaussians with zero means and shared orientations may be more suitable. In this case, we parameterize an anisotropic zero-mean Gaussian as:

$$\begin{array}{c} G_{w,\sigma_{x},\sigma_{y},\alpha }\left(x,y\right)=w\;\text{exp}\left(-ax^{2}-2bxy-cy^{2}\right)\; \end{array}$$ (2)

where for an orientation angle *α* ∈ [0; *π*) the coefficients *a*, *b*, *c* are defined as:

$$\begin{array}{c} a=\frac{\cos^{2}\alpha }{2\sigma_{x}^{2}}+\frac{\sin^{2}\alpha }{2\sigma_{y}^{2}} \end{array}$$ (3)

$$\begin{array}{c} b=-\frac{\sin2\alpha }{4\sigma_{x}^{2}}+\frac{\sin2\alpha }{4\sigma_{y}^{2}} \end{array}$$ (4)

$$\begin{array}{c} c=\frac{\sin^{2}\alpha }{2\sigma_{x}^{2}}+\frac{\cos^{2}\alpha }{2\sigma_{y}^{2}} \end{array}$$ (5)

The anisotropic DoG filter is then defined as:

$$\begin{array}{c} \text{DoG}\;\text{Ansotropic}\left[w_{1},w_{2},\sigma_{1,x},\sigma_{1,y},\sigma_{2,x},\sigma_{2,y},\alpha \right]\left(x,y\right)=\\ G_{w_{1},\sigma_{1,x},\sigma_{1,y},\alpha }-G_{w_{2},\sigma_{2,x},\sigma_{2,y},\alpha } \end{array}$$ (6)

We refer to the DoG filter (6) as the *Anisotropic* or *seven-parameter DoG filter* based on the number of associated parameters. The *five-parameter DoG filter* can be obtained by fixing the constants *w*_1_ and *w*_2_ to be normalizing, i.e. $w_{i}=1/2\pi \sqrt{\sigma_{i,x}\sigma_{i,y}}$. An example of anisotropic Difference of Gaussians is depicted in Fig 2(c). The usage of anisotropic difference of Gaussians allows detecting different kinds of elongated blobs with only three additional trainable parameters per filter (compared to the two- or four-parameter versions).

Overall, DoG filters provide a simple way to parameterize blob-detecting linear filters using a small number of parameters. They can also be extended to three-dimensional blob detection in a straightforward manner. Since in three dimensions generic linear filters come with an even larger number of parameters, the use of DoG parameterization is even better justified. Here, one natural choice would be to use differences of Gaussian filters that are isotropic within axial slices and use a different variance (bandwidth) along the axial dimensions:

$$\begin{array}{c} G_{w,\sigma ,\sigma_{z}}\left(x,y,z\right)=w\;\text{exp}\left(-\frac{x^{2}+y^{2}}{2\sigma^{2}}-\frac{z^{2}}{2\sigma_{z}^{2}}\right) \end{array}$$ (7)

$$\begin{array}{c} \text{DoG}\;\text{3D}\left[w_{1},w_{2},\sigma_{1},\sigma_{2},\sigma_{1,z},\sigma_{2,z}\right]=G_{w_{1},\sigma_{1},\sigma_{1,z}}-G_{w_{2},\sigma_{2},\sigma_{2,z}} \end{array}$$ (8)

Generally, as axial resolution in 3D fluorescence microscopy is typically lower, *σ*_*i*,*z*_ is also taken to be larger than *σ*_*i*_. The filter (8) provides a six-parameter parameterization of a family of 3D blob detection filters (one of which is visualized in Fig 2(d)), whereas a generic 3D filter takes *O*(*d*^3^) parameters, where *d* is the spatial window size.

### “Shallow” DoGNet

The shallow (single layer) Difference of Gaussians network (DoGNet) is a neural network built around DoG filters Fig 2(a). It takes as an input a multiplexed fluorescence image, applies multiple DoG filters (1),(6) or (8) to each of the input channels. Subsequently, DoGNet combines the obtained maps linearly (which in deep learning terminology corresponds to applying 1 × 1 convolution). The latter step obtains a single map of the same spatial resolution as the input image. Finally, a sigmoid non-linearity is applied to convert the applied maps into probability maps.

More formally, we define a single-layer DoGNet as

$$\begin{array}{c} \Psi \left(X;\theta =\left\{\gamma ,\beta ,\zeta \right\}\right)=S\left(\left(X⊛DoG_{\beta }\right)⊛\gamma +\zeta \right), \end{array}$$ (9)

where *X* denotes the input multiplexed image, ⊛ is the 2D convolution operation, and the vector *β* denotes the parameters of all DoG filters. Assuming that the input contains *N* channels, and each channel is filtered with *M* DoG filters, the application of all DoG results in *M* × *N* maps. Those maps are then combined into *K* maps using a pixel-wise linear operation (which can be treated as a convolution with 1 × 1 filters). The tensor corresponding to such linear combination and containing *K* × *M* × *N* values is denoted *γ*.

To each of the obtained *K* maps, the bias value *ζ*_*k*_ is added, and finally all obtained values are passed through the element-wise sigmoid non-linearity *S*(*x*) = 1/(1 + exp(−*x*)). Overall, *θ* in (9) denotes all learnable parameters of the DoGNet.

In the case of the single-layer DoGNet, the output has a single map (i.e. *K* = 1). Except for the last sigmoid operation, the single-layer DoGNet contains only linear operations and can be regarded as a special parameterization of the linear filtering operator that maps the input *M* maps to several output maps, usually two maps.

### Deep DoGNet

The deep DoGNet architecture is obtained simply by stacking multiple DoGNet layers (9):

$$\begin{array}{c} \Phi \left(X;\theta =\left\{\theta_{1}\ldots \theta_{T}\right\}\right)=\Psi \left(\Psi \left(\ldots \Psi \left(X,\theta_{1}\right)\ldots ;\theta_{T-1}\right);\theta_{T}\right), \end{array}$$ (10)

where *T* is the number of stacked single layers DoGNets, and *θ*_*t*_ denotes the learnable parameters of the *t*-th layer. The final number of maps *K*_*T*_ is once again set to one, so that the whole network outputs a single probability map. However, the numbers of layers *K*_*t*_ that are output by the intermediate DoGNet layers would typically be greater than one. In our experiments the number of sequential layers *T* was set to three.

### Element-wise multiplication

Inspired by an idea from [32], instead of producing a single probability map, our network delivers two independent maps and using the element-wise product of those maps we get the final map. We have implemented this approach as a separate layer and that does not require any trainable parameters. In the case of synapses, this step allows reducing the effect of displacement between pre- and postsynaptic punctae by learning probability maps independently for pre and postsynaptic signals. Given several probability maps (for pre- and postsynaptic punctae) the element-wise products will act as a logical operator “AND,” highlighting the intersection between those maps, where the synaptic cleft is located. In our research we use element-wise multiplication not only for DoGNets but for baselines as well, they all benefit from this layers.

### DoGNet initialization

We have found that appropriate parameter initialization is key to obtaining reproducible results with our approach. Popular neural networks have a redundant number of parameters and are initialized by sampling their values from a Gaussian distribution. This initialization is not suitable for DoGNets because of the relatively small number of parameters. Instead, we use a strategy from object detection frameworks [34]. This approach consists of initialization with a range of reasonable states (priors). An optimization procedure selects the best priors and tunes their parameters. In DoGNet we use Laplacian of Gaussians with different sizes that are sampled from a regular grid as priors. Specifically, we obtain the Gaussian variance (*sigma*) by splitting the line segment [0.5, 2] into equal parts. The number of parts depends on the number of DoGs reserved for each image plane (in our experiments that number was set to five). We set the difference-variance in the Laplacian of Gaussians to 0.01. For example, if we set the number of DoGs for a channel to 3, the sigmas will be 0.5, 1.25, and 2, respectively.

### Training DoGNets

We train the described architecture by minimizing the *softdice* loss (11) proposed in [35] between the predicted probability map Ψ(*X*; *θ*) and a ground truth mask *Y*_*g*_:

$$\begin{array}{c} L_{\theta }\left(X,Y_{g}\right)=1-2\frac{\sum Y_{g}\Psi \left(X;\theta \right)}{\sum {\Psi (X;\theta )}^{2}+\sum {Y_{g}}^{2}} \end{array}$$ (11)

Here, sums are taken over individual pixels, and in the ground-truth map *Y*_*g*_ all pixels belonging to synapses are marked with ones, while the background pixels are marked with zeros. In the experiments we found that on the imbalanced data typical for synapse detection problems, this loss performs better than standard binary cross entropy.

In order to optimize this loss function, partial derivatives with respect to DoGNet parameters *dL*/*dθ* must be obtained, which may be accomplished via backpropagation [33]. The backpropagation process computes the partial derivatives with respect to the filter parameters at each of the spatial positions within the spatial support of the filter (which we limit to 15 pixels). The partial derivatives with respect to the DoGNet parameters are then obtained by differentiating formulas (1),(6) or (8) at each spatial location and multiplying by the respective derivatives.

The ground truth mask *Y*_*g*_ as well as the input images *X* for the training process are obtained using a combination of manual annotation and artificial augmentation. The synapse detection in FM images is a challenging and arguably ambiguous task even for human experts. Furthermore, even a small, 100 × 100 pixel region of an image might contain more than 80 synapses. In practice it is impossible to annotate the borders of each synapse accurately, therefore the experts were asked to mark the centroid of synapses only, corresponding to the synaptic cleft, after which all pixels within a radius of 0.8*μm* were assigned to the corresponding synapse. We trained DoGNets for 5000 epochs. Each epoch is a set of ten randomly cropped subsamples 64 × 64 from the annotated training dataset. Because DoGNets have few parameters, we found that the training processes converged rapidly typically requiring only several minutes on an NVidia Titan-X GPU for the datasets described below. Once trained, inference can be performed on a CPU as well as on a GPU using the implementations of Gaussian filtering that may be optimized for a particular computing architecture. Our implementation uses the PyTorch deep learning framework [36], which allows for concise code and benefits from automatic differentiation routines.

### Post-processing

Because both shallow and deep versions of DoGNet produce probability maps rather than lists of synapse locations and parameters, these probability maps need to be postprocessed in order to identify synapse locations and properties. Toward this end, first, we reject points with low confidence by truncating the probability maps using a threshold of *τ* of 0.5. In order to extract synapse locations from the probability map produced by the DoGNet, we need to find local maxima. In standard fashion, we greedily pick local maxima in the probability map, traversing them in the order of decreasing probability values while suppressing all maxima within a cut-off radius *R* = 1.6*μm* from previously identified maxima (so called *non-maxima suppression*) [37]. The output of this procedure is the *x* and *y* locations of synaptic puncta.

The next step is to describe each detected punctum with a vector containing the information about the detected synapse. To obtain a descriptor for a synapse, we select a small window of the same radius *R* = 1.6*μm* around its location, fit Gaussian distributions to each of the input channels, and for each protein marker we store the average intensity, the displacement of the Gaussian mean with respect to the window center, the Gaussian orientation, and its asymmetry. Evaluating the quality of such a descriptor is left for future work.

## Results

### Datasets

The proposed method and a set of baselines were evaluated on four independent datasets for which synapses were annotated manually: *[Collman15]* dataset of conjugate array tomography (cAT) images [16], *[Weiler14]* dataset of array tomography (AT) images [17], *[PRISM]* dataset of multiplexed confocal microscopy images [6], and a synthetic dataset that we generate here. Each published experimental dataset was obtained using fluorescence imaging based on commercially available antibodies, with synapsin, vGlut, and PSD-95 markers common to the datasets. At the end of section, we additionally perform comparisons using synthetic dataset with excitatory and inhibitory synapse sub-types.

### Compared methods

In each of our trials we compared several DoGNet configurations with several baseline methods including reduced version of the fully convolutional network (FCN) [15], and an encoder-decoder network with skip connections (U-net) [8]. An exhaustive comparison between different deep architectures is a nearly impossible task, mostly because of an infinite number of possible configurations. Nevertheless, we have done our best to tune the parameters of the baseline methods. The best-performing variants of the baseline architectures (FCN, Unet) were used in the experiments and are described in detail in the supplementary material. To make our evaluation more direct, we have designed the competitive networks to have the same receptive field (FOV) (arbitrarily chosen to 15 pixels). We have also evaluated two *manually-tuned* methods, namely the probabilistic synapse detection method [32] and the image processing pipeline proposed in [38]. Detailed technical background on these architectures are described in supplementary materials.

The DoGNet architecture has two major options: Shallow and Deep, with the Shallow option corresponding to a single layer and the Deep option corresponding to number of sequential layers. The second word in our notation *Isotropic* or *Anisotropic* indicates the number of degrees of freedom in the DoG parameterization, e.g. Isotropic denotes four-degree DoG (1). The number of DoG filters for each channel was arbitrary set to five. We also evaluated a simple ablation denoted as *Direct* that takes the Shallow Isotropic DoGNet architecture and replaces DoG-parameterized filters with 15 × 15 unconstrained filters (thus using Direct parameterization)(see Supplementary Information).

### Error metrics

The quality of synapse detection was estimated using the standard metrics: precision, recall, and F1-score, with the output of each method consisting of the set of points denoting synapse coordinates. True positives were estimated as the number of paired points between annotation and detection provided the distance between them was less than half of the mean synapse radius (*ρ* = 0.6*μm*). To avoid multiple detections of synapses (false positives), we require that each detected point can be matched at most once. Detections and annotations without pairs were considered to be false positives and false negatives, respectively. The *precision* measure was then computed as the ratio of true positives to all positives, and the *recall* measure as the ratio of true positives to all synapses contained in the annotation. The F1-score combines the precision and recall in one criterion by taking the double product of recall and precision divided over their sum. For evaluation purposes, we also added the AUC criterion corresponding to the area under the ROC curve obtained by varying the confidence threshold *τ*. This criterion is stable to the threshold choice and depends on the quality of the probability map produced by a method. For different thresholds, we estimated the conjunctions between probability map and ground truth binary segmentation pixel-wise.

For quantitative comparison, we have also used the absolute difference in counting (|*DiC*|). This metric merely computes the difference between the number of synapses detected using a method and the ground truth. This measure does not answer the question of how well a synapse was localized but still gives additional insight into quantitative results.

Since the training procedure is a probabilistic process depending on initialization and data sampling, we estimate each value as the mean of five independent runs.

### Results on PRISM dataset

To verify our method on PRISM data [6], we performed manual dense annotation of several image regions of a dataset of FM images obtained using this technique. The manual annotation was performed by two experts using synapsin, vGlut, Bassoon and PSD-95 channels. Each expert annotated three regions. The total set was made of six regions and split into training, validation (392 synaptic locations) and testing subsets (173 synaptic locations). Each subset consisted of two regions annotated by different experts, with test regions overlapped in order to estimate inter-expert agreement. For synapse annotation, we developed a graphical user interface. This software allows selecting image channels and regions. As we solve the task of semantic segmentation during the training, we need a densely annotated image region. We mark each synapse with a point approximately at the synaptic cleft.

Evaluation against baselines is presented in Table 1. Due to circular puncta shape and the relatively small displacement of markers, the optimal method was Shallow Anisotropic with only 107 trainable parameters. This configuration also performed considerably better than the Direct Ablation approach, highlighting the advantage of using DoG parameterization in place of direct parameterization of the filters.

**Table 1:** Comparison of several variations of DoGNets and several baselines on PRISM dataset.

Method	# params	F1 Score	Precision	Recall	AUC	|*DiC*|
**ConvNets**						
Direct	3392	0.74	0.66	0.84	0.85	17.67
FCN	3002	0.75	0.73	0.77	0.84	7.44
Unet	622	0.80	0.78	0.83	0.88	10.44
**DoGNets**						
Shallow Isotropic	62	0.78	0.72	0.87	0.91	15.22
Shallow Anisotropic	107	**0.83**	0.81	0.86	0.91	4.89
Deep Isotropic	140	0.81	0.81	0.82	0.89	9.78
Deep Anisotropic	230	0.80	0.81	0.80	0.83	7.89
**Manually tuned methods**						
Nieland 2014 [38]	-	0.78	0.72	0.84	0.82	1.
Simhal 2017 [32]	-	0.50	0.45	0.58	0.68	21.

We performed several analyses in order to evaluate agreement between three independent human experts as well as between the experts and our method (Table 2). Importantly, the proposed network agreed with the Experts similarly to the agreement between the Experts themselves.

**Table 2:** Agreement between DoGNet and three independent human experts on the task of synapse detection on the PRISM dataset.

Trial	F1 Score	Precision	Recall
Shallow Isotropic vs Expert 1	0.83	0.83	0.84
Shallow Isotropic vs Expert 2	0.87	0.91	0.83
Shallow Isotropic vs Expert 3	0.86	0.90	0.83
Expert 1 vs Expert 2	0.82	0.86	0.78
Expert 3 vs Expert 2	0.81	0.81	0.8
Expert 3 vs Expert 1	0.77	0.79	0.8

### Results on Collman15 dataset

**Fig 3:**
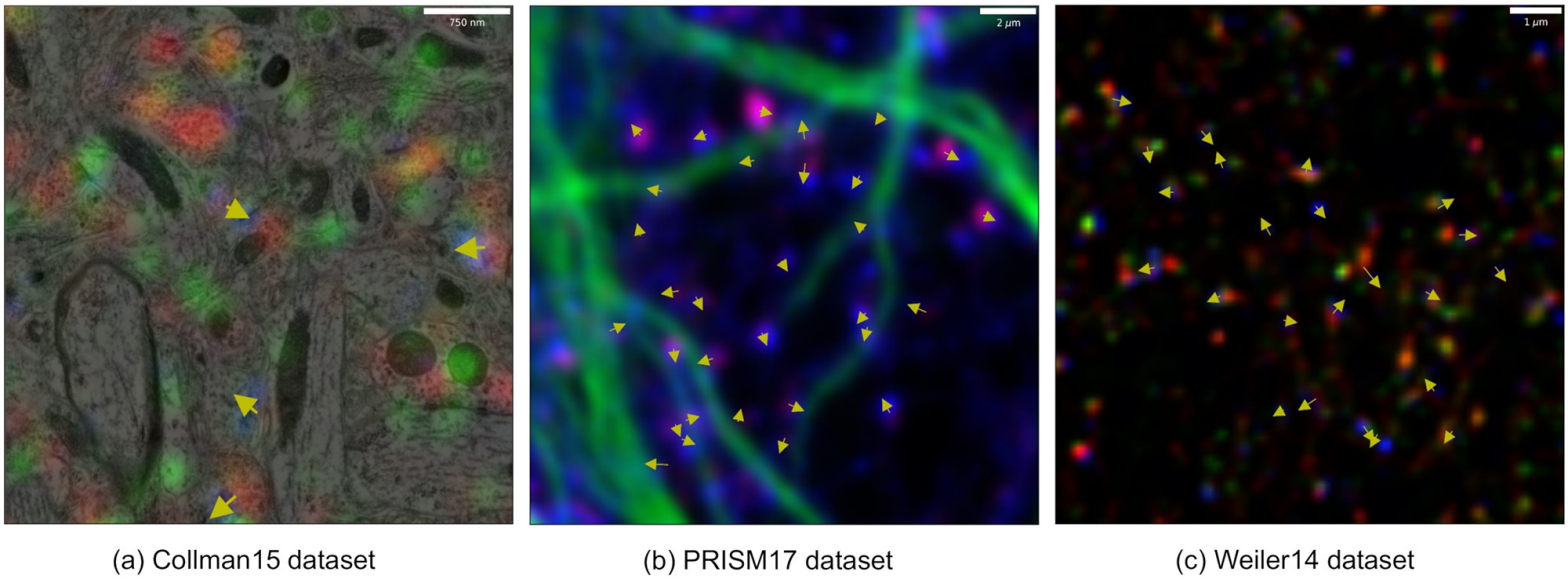
Results of DoGNet synapse detection on distinct datasets. Yellow arrows denote synapse orientation from presynaptic to postsynaptic sides. (a) The Collman15 dataset is a mixture of EM and FM images (EM is shown in grayscale, the red, green, and blue channels show the intensity of synapsin, vGlut, and PSD95 respectively). (b) The PRISM dataset. False color scheme has red channel corresponding to synapsin, blue to PSD95, and green to the cytoskeletal marker MAP2, which indicates how synapses are distributed along microtubules. (c) The Weiler14 dataset. The red, green, and blue channels show the intensity of synapsin, vGlut, and PSD95, respectively.

In this dataset, the alignment of electron microscopy (EM) and array tomography (AT) images provides the ground truth for synapse detection using fluorescence markers. Using high resolution EM data synaptic clefts and pre- versus post- synaptic sites can be identified unambiguously, which was used as validation for the synapse detections from fluorescence data (Fig 3(a)). The dataset contains 27 slices of 6310 × 4520 pixels each, with a resolution of 2.23 × 2.23 × 70 *nm*, and contains annotation with pixel-level segmentation of synaptic clefts. In order to fit our training procedure, we have used only synaptic cleft centroid coordinates. The EM resolution is much greater, so AT data were interpolated to be aligned with EM data. Provided we utilize solely AT data, its original resolution of 0.1*μm* per pixel can be recovered without losing any information. The first five slices were used as the train dataset, whereas the remainder (slices 6-27) served as the test dataset.

Results of our evaluation (Table 3) show that shallow DoGNets exhibit highest performance in terms of the F1-measure. The receptive field 15 × 15 pixels followed by inter-channel element-wise multiplication allow capturing highly displaced markers puncta combinations. Displacements in marker punctae occur because synapses are 3D objects with random orientations. Therefore, the presynaptic and postsynaptic signals in the image plane produce displaced peaks up to a half of a micron. The closest-performing ConvNet architecture was U-net with 622 trainable parameters; increasing the number of its parameters led to overfitting and therefore lower performance on the test dataset examined here.

**Table 3:** Comparison of several variations of DoGNets and several baselines on the [Collman15] dataset. The ‘Shallow3D’ network uses the 3D version of DoGNet, while other variants operate on 2D slices independently. Optimal performance was obtained using Shallow DoGNets.

Method	params	F1 Score	Precision	Recall	AUC	|*DiC*|
**ConvNets**						
Direct	3392	0.69	0.79	0.62	0.88	11.19
FCN	3002	0.71	0.72	0.70	0.79	4.12
Unet	622	0.73	0.73	0.73	0.91	4.26
**DoGNets**						
Shallow Isotropic	62	**0.75**	0.74	0.76	0.90	4.25
Shallow Anisotropic	107	**0.75**	0.75	0.76	0.88	4.26
Shallow3D	61	0.68	0.62	0.77	0.65	9.13
Deep Isotropic	140	0.73	0.77	0.71	0.97	4.99
Deep Anisotropic	230	0.71	0.77	0.33	0.87	7.72
**Manually tuned methods**						
Nieland 2014 [38]	-	0.37	0.49	0.32	0.63	16.5
Simhal 2017 [32]	-	0.65	0.52	**0.89**	0.74	-

The AT stains include markers specific for excitatory (vGlut, PSD95) and inhibitory (GABAergic, gephyrin) synapses. In our experiments, the use of inhibitory markers did not improve the detection scores. Moreover, the precision of all trainable methods was considerably lower using only inhibitory markers (synapsin, GABA, gephyrin).

### Results on Weiler dataset

The Weiler dataset [17] consists of 12 different neural tissue samples. Each sample was stained with a number of distinct antibodies including synapsin vGlut, and PSD-95. For each stain, 70 aligned slices were acquired using array tomography (AT). Each slice was a 3164 × 1971 pixel image with spatial resolution of 0.2*μm* per-pixel. This dataset does not have any published annotation.

We investigated the ability of DoGNets to generalize across distinct datasets by applying networks trained on the well-annotated [Collman15] dataset, which was annotated using serial electron microscopy data, to the previously unlabeled AT dataset [Weiler14] [17]. Generally, the staining of [17] is similar to the Collman15 dataset [16]. Thus, we first performed a coarse alignment by resizing [Collman15] images and applying linear transforms to the intensities of each channel so that the magnification factors, means, and standard deviations of the intensity distributions were matched. The architectures trained on [Collman15] were then evaluated on [Weiler14].

Qualitative examples of this cross-dataset transfer are shown in Fig 3. For quantitative validation we generated manual annotations of two randomly selected regions of the [Weiler14] dataset using the same software that we have used for [PRISM] annotation. We observed that the levels of agreement between the results of the DoGNet Shallow Anisotropic trained on [Collman15] dataset and each of the experts were similar to the level of inter-expert agreement (in terms of the F1 score).

The results of this cross-dataset validation are shown in Table 4. Importantly, while the performance of compared methods, did not diminish dramatically. In fact, the DoGNets actually improved in their performance, which we attribute to the fact that in the Weiler dataset all expert annotations were based on FM images, rendering the analysis more straightforward in comparison with the [Collman15] synapses that are visible in EM data but not in the FM data that were not included.

**Table 4:** The quantitative validation of DoGNet trained on [Collman15] cAT dataset and applied to [Weiler14] dataset. Differences with F1 scores on [Collman15] cAT dataset are shown in parentheses.

Method	# params	F1 Score	Prec.	Recall	AUC	|*DiC*|
**ConvNets**						
Direct	3392	0.72 (0.03)↑	0.79	0.66	0.88	5.33
FCN	3002	0.64 (-0.07)↓	0.85	0.51	0.84	19.
Unet	622	0.79 (0.06)↑	0.85	0.74	0.97	4.33
**DoGNets**						
Shallow Isotropic	62	0.85 (0.1)↑	0.83	0.88	0.96	3.33
Shallow Anisotropic	107	0.83 (0.08)↑	0.88	0.78	0.94	3.33
Deep Isotropic	140	**0.88** (0.15)↑	0.83	0.95	0.93	3.33
Deep Anisotropic	230	0.71 (0.0)	0.80	0.63	0.90	7.33
**Manually tuned methods**						
Nieland 2014 [38]	-	0.64 (0.27)↑	0.66	0.62	0.44	2.
Simhal 2017 [32]	-	0.65 (0.0)	0.81	0.55	0.55	13.

### Synthetic dataset

In order to further evaluate our approach rigorously in a fully controlled setting, we also applied it to a synthetic dataset. The goal of the evaluation of DoGNet using synthetic data was to estimate the quality of synapse detection compared with baseline procedures for distinct levels of signal-to-noise ratio; including the presence of spurious synapses; and for different presynaptic-to-postsynaptic markers displacements on image planes to emulate the 3D structure of synapse. Further, this systematic evaluation using synthetic data addresses questions regarding meta-parameter choice, methodological limitations, and the justification of neural network usage for synapse detection tasks. Because the number of training samples was unlimited, deep networks with a large number of parameters were unlikely to overfit the data.

Our dataset models three entities: true synapses, spurious synapses that emulates false bindings, and random noise. We emulated true synapses and spurious synapses using Gaussian probability density functions placed in different image planes with additive white noise, where each image plane refers to a specific protein marker such as synapsin, vGlut, PSD-95, vGat or gephyrin. To assess the generalized performance of different architectures, in our synthetic experiments we simulated both excitatory and inhibitory synapses.

Spurious synapses are made to emulate false bindings in combination with random noise in order to act as a distraction for the classifier to evaluate its robustness. An actual synapse has intensity peaks at least in one presynaptic and in one postsynaptic image plane, while spurious synapses have peaks only in presynaptic or postsynaptic channels, but never in both. An example of a true excitatory synapse might be a signal that has a punctum in synapsin, vGlut and PSD-95 markers separated by a distance less than a half of a micron. An inhibitory synapse would have punctae in synapsin, vGat and gephyrin. The displacement in markers punctae, caused by the 3D structure of synapses, makes the process of differentiation between actual and spurious synapses considerably more challenging, thereby rendering the simulation more realistic. The intensity of the synaptic signal were emulated using Log-Normal distribution with zero mean and *σ* = 0.1.

**Fig 4:**
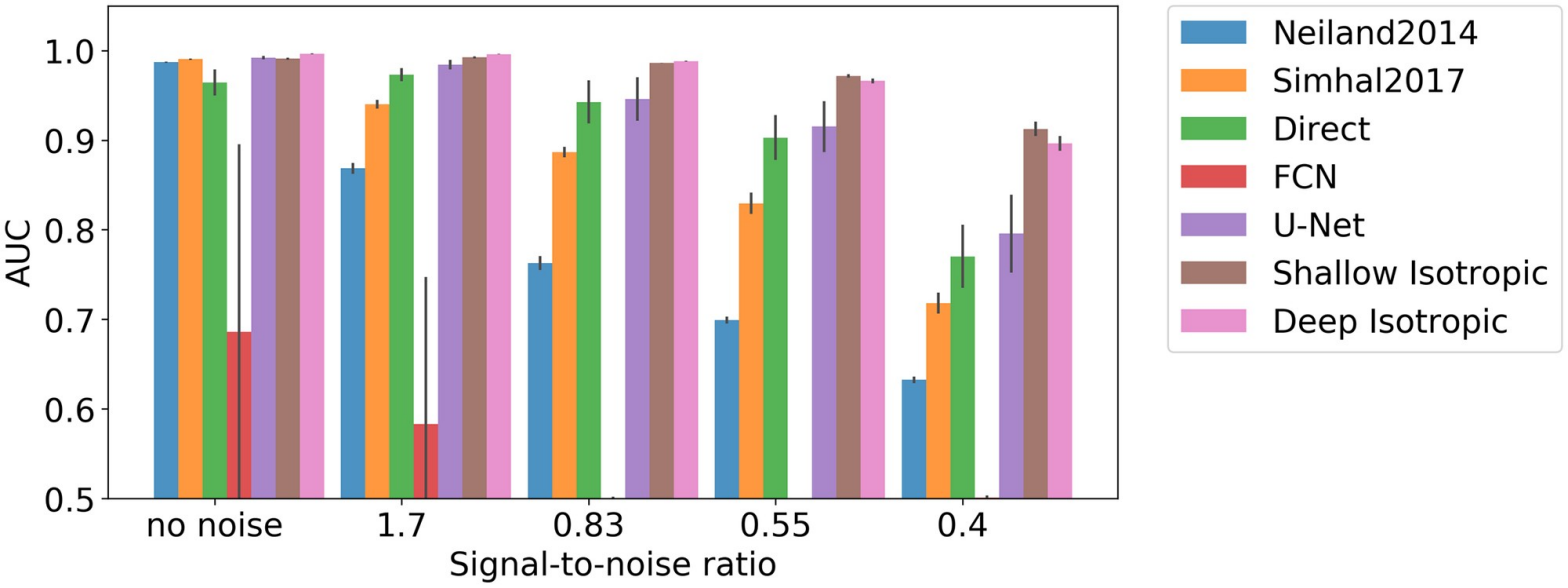
Method sensitivity to signal-to-noise ratio. A comparison of manually-tuned methods, deep architecture baselines, and DoGNets. The bar chart shows differences in methods in area-under-curve (AUC) measure for different signal-to-noise ratios. DoGNets are more robust to noise than manually tuned methods, with low variation in AUC between DoGNet runs.

**Fig 5:**
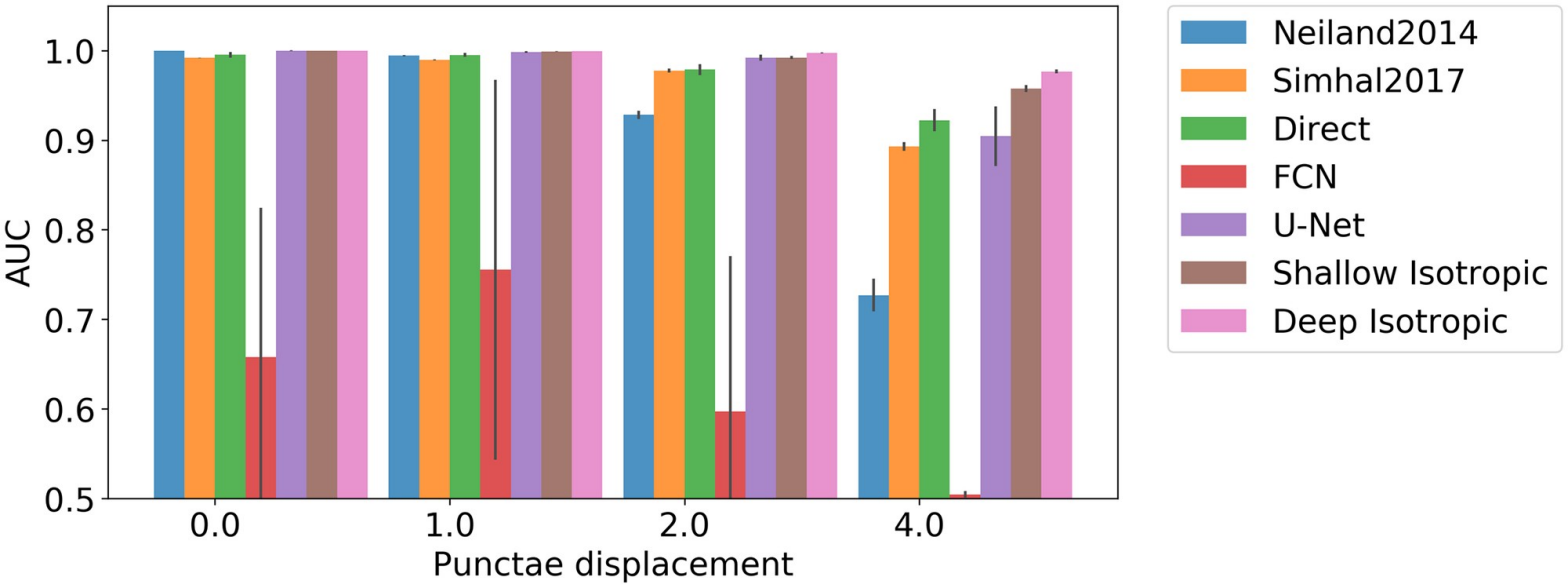
Methods sensitivity to punctae displacement. With increasing displacement, it is more difficult to discriminate between true synapses and spurious synapses. The quality of the segmentation map produced by DoGNets decreases more slowly than that of other methods.

Modeling synapses using isotropic Gaussians in our synthetic dataset enables the initial evaluation of purely isotropic DoGNets. First the sensitivity of the approach to signal-to-noise ratio was evaluated (Fig 4). Results indicate that small convolutional neural networks are sensitive to initialization and may become trapped in local minima, whereas DoGNet performance was more robust, although DoGNets initialized randomly rather than using our initialization scheme also suffered from local minima. Importantly, deeper architectures were capable of handling larger displacements between punctae (Fig 5). This result is anticipated because multi-layer architectures have larger receptive fields and capture more non-linearities, allowing the capture of more complex relations in the data. For example, in the presence of substantial displacements, at least one additional convolution layer followed by an element-wise multiplication was needed to perform a logical AND operation between pre and post synaptic channels after blob detection [32].

**Fig 6:**
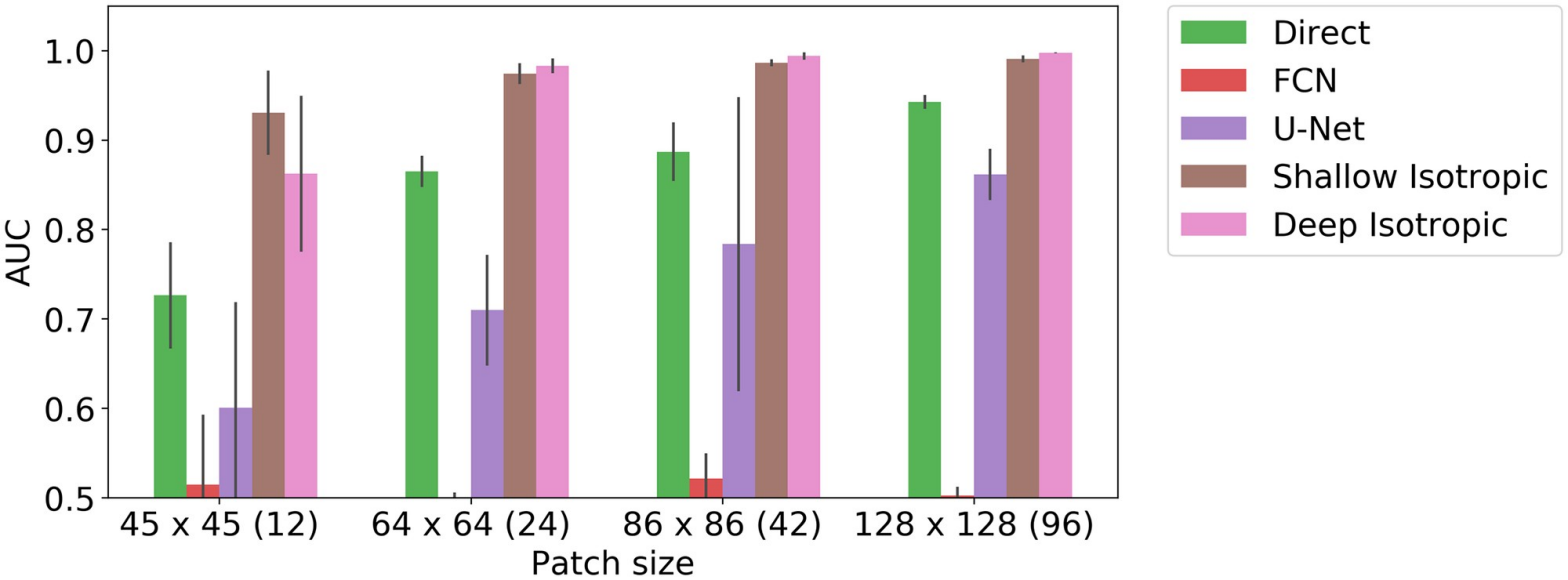
Methods sensitivity to small numbers of training examples. Comparison of different trainable architecture baselines with DoGNets for various amount of training data. In this experiment, the training sets corresponded to patches of different sizes, ranging from 45 × 45 pixels (with approximately 12 synapses) to 128 × 128 (with approximately 96 synapses). The maximal displacement was set to two pixels, the signal-to-noise ratio was fixed to 3.0, and no augmentation such as random cropping was applied. Shallow DoGNets need only few examples to reach acceptable performance. With a sufficient number of examples the baseline architecture can perform as well as or better than DoGNets.

We also present a study of training with limited examples. We have evaluated trainable methods (Direct, FCN, U-Net, Shallow Isotropic, Deep Isotropic) on fixed size crop without any augmentation in search of minimal size of image region when each method starts work suitable the signal-to-noise ration was sent to approx 4.5 and the maximal displacement to two pixels. We present the results of this study in (Fig 6). We show that Shallow and Deep DoGNets are able to learn a simple signal like a multiplexed blob form only few samples.

## Discussion

We introduce an efficient architecture (DoGNet) for the automatic detection of neuronal synapses in both cultured primary neurons and brain tissue slices from multiplexed fluorescence images. Under some conditions, the accuracy of DoGNet accuracy approaches the level of agreement between human annotations. DoGNet also outperforms ConvNets when the number of training examples is limited. Importantly, the DoGNet approach is capable of efficiently integrating a number of different input images from multiplexed microscopy data with a larger number of channels, which can be prohibitively difficult for human experts to accomplish efficiently. This allows for the detection of synapses in large datasets and facilitates downstream quantitative analysis of synaptic features including brightness or intensity, size, and asymmetry.

The robust automated detection of synapses is important for downstream synapse classification, particularly as multiplexed imaging modalities such as PRISM are applied to larger-scale genetic and compound screens, which rely on phenotypic classification of synapses to understand the molecular basis of neurological diseases. By integrating features of synapses detected using machine learning techniques, the proposed method can be used to classify synapses to study their identities and spatial distributions. In conjunction with dendrite and axon tracking [39], this approach may be used to build connectivity maps, tracing synaptic connections for each individual neuron.

DoGNet is computationally efficient during both training and inference. Training the simplest model Simple Isotropic required only 7.37 seconds on an NVidia TitanX GPU and 37.84 seconds on Intel i7 CPU for 2000 epochs, which is several times faster than training U-Net and FCN ConvNets. Each epoch is an array of ten patches 64 × 64 pixels randomly cropped from the training set. The inference process for a 1000 × 1000 image requires only 0.001 second on a Titan-X GPU and only 0.1 second on Intel i7 CPU. Most of this time is consumed by post-processing, making it suitable for both high-throughput studies and small-scale experiments without GPU acceleration. The proposed architecture is not specific to synaptic images, and can be applied to other cellular or tissue features where objects of interest show punctate spatial patterning, such as single molecule annotation in super-resolution imaging and single-particle tracking, detection of exocytic vesicles, and detection of puncta in mRNA FISH and in situ sequencing datasets [20, 21]. In cases where high precision estimates of puncta features, such as their spatial extent and centroid positions exists, it may be beneficial to follow DoGNet segmentation with dedicated point spread function (PSF) fitting methods such as Maximum Likelihood Estimation or Least Squares fitting. In this case, DoGNet could be used to improve and streamline initial segmentation tasks that generally occur prior to more robust PSF fitting methods in analysis pipelines [40, 41].

Despite the preceding strengths, the proposed method also has several limitations, most of which are common to supervised methods. First, DoGNet is useful for synapses because synapse sizes are on the order of the resolution of the light microscope, and thus present as diffraction limited spots. However, this approach would be unsuitable to more complex, larger objects such as nuclei, bacterial cells, or possibly large organelles. In summary, DoGNets are limited to the class of 2D signals with a convex shape and limited radius (blobs). A second limitation is the dependency on the proper parameter initialization scheme. For DoGNets, which have fewer parameters, improper initialization of a single parameter, for example setting *σ* close to zero, can cause the entire network to diverge. In contrast, ConvNets with a larger number of parameters can more easily recover from improper initialization. Notwithstanding, we have found that our initialization scheme for DoGNets works reliably across multiple runs and distinct datasets. For practical use, shallow DoGNet seems to be more reliable than deep DoGNets. We note that shallow DoGNet can still become a part of more complex networks.

We have also shown the ability of DoGNets to transfer across datasets by training them on one AT dataset [*Collman15*] and applying them to another, distinct dataset [*Weiler14*]. This type of transfer may prove useful in the detection of synapses with high confidence by training DoGNet on either cAT data sets such as [*Collman15*] [16] or highly multiplexed datasets such as [*PRISM*] [6], which are more difficult to acquire experimentally but facilitate synapse annotation with higher certainty. Specifically, electron microscopy allows for highly robust synaptic annotation through conserved features of the synaptic cleft and the post-synaptic density, whereas multiplexed fluorescence data allow for accurate annotation of synapses through the colocalization of multiple synaptic markers.

### Conclusion

We present DoGNet—a new architecture for blob detection. While DoGNets are applied here to synapse detection in multiplexed fluorescence and electron microscopy datasets, they are more broadly applicable to other blob detection tasks in biomedical image analysis.

Due to their low number of parameters, DoGNets can be trained in a matter of minutes, and are suitable for non-GPU architectures because the application of a pretrained DoGNet amounts to a sequence of Gaussian filtering and elementwise operations. In our experiments, DoGNets were able to robustly detect millions of synapses within several minutes in a fully automated manner, with accuracy comparable to human annotations. This computational efficiency and robustness may prove essential for the application of multiplexed imaging to high-throughput experimentation including genetic and drug screens of neuronal and other cellular systems.

## Supporting information

S1 Text Baseline network architectures. (PDF)

S1 Fig Results of Shallow Isotropic DoGNet on PRISM dataset. The top image is the original one, the middle is the probability map produced by DoGNet and on the bottom is the overlay of detected synapses on the original image. Detected synapses are denoted with a red arrow, indicating their orientation concerning pre- and postsynaptic sides. The ground truth synapses locations are depicted using white crosses. Yellow bounding box highlights the densely annotated region.
(TIF)

S2 Fig Results of Deep Anisotropic DoGNet on the Weiler14 dataset. (TIF)

S3 Fig Results of Deep Isotropic DoGNet on the Collman15 dataset. (TIF)
