# Supplementary material for: DoGNet: A deep architecture for synapse detection in multiplexed fluorescence images
Source: PLoS Comput Biol. 2019 May 13;15(5):e1007012. doi: 10.1371/journal.pcbi.1007012 (PMC6533009; doi:10.1371/journal.pcbi.1007012)

Baseline networks

Direct network. The direct network is a copy of Shallow DoGNet, where each element of the convolution filter is learned independently. The first convolution takes as input n channels and convolves each with five filters of size 15×15 pixels. Next 5n channels are linearly weighted and converted into two independent probability maps by a sigmoid function. To get the resulting probability map, we multiply them element-wise.

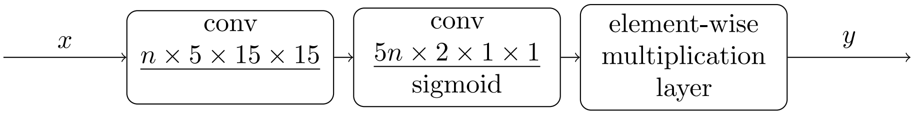

Fully convolutional network. Fully convolutional network (FCN) is a stacking of multiple convolutional layers with non-linearity.

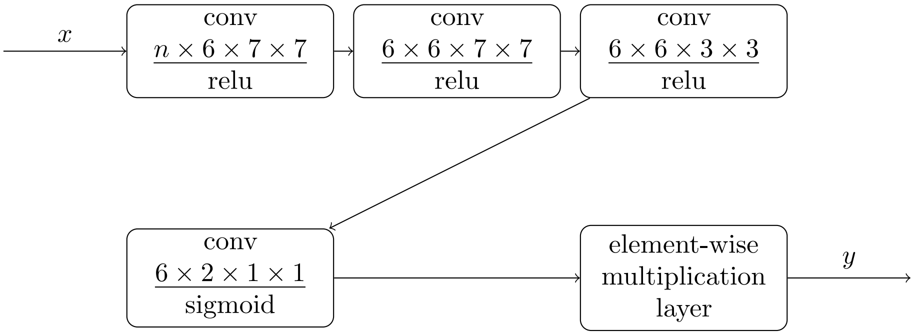

U-net. U-net is a standard baseline in microscopy analysis task. Do to a redundant number of parameters of the original u-net; we have implemented a minimalist version. The minimalist U-net, used in our work, was made of two scales and composed of five convolution operations.

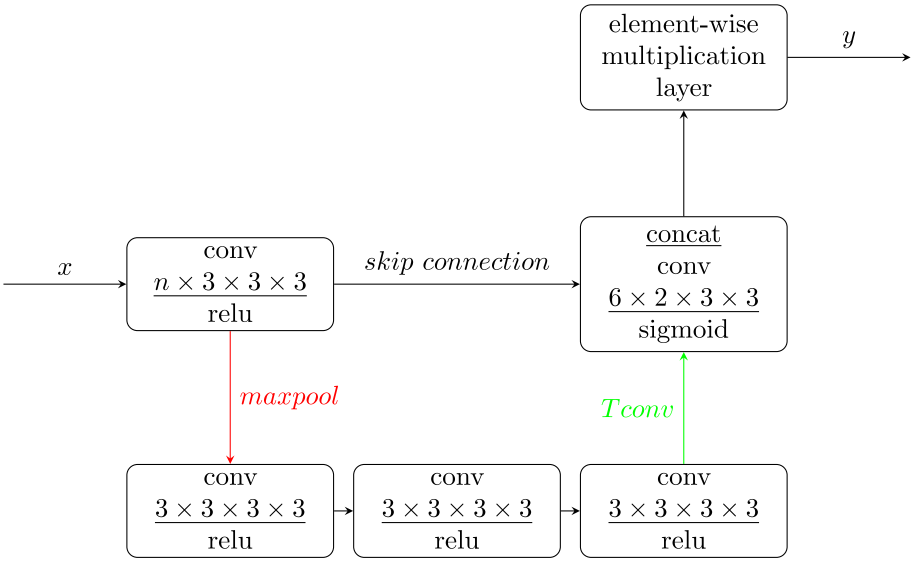

Supplement: S1 Text — (PDF) [file pcbi.1007012.s001.pdf]
