# Supplementary figures and images for: DoGNet: A deep architecture for synapse detection in multiplexed fluorescence images
Source: PLoS Comput Biol. 2019 May 13;15(5):e1007012. doi: 10.1371/journal.pcbi.1007012 (PMC6533009; doi:10.1371/journal.pcbi.1007012)

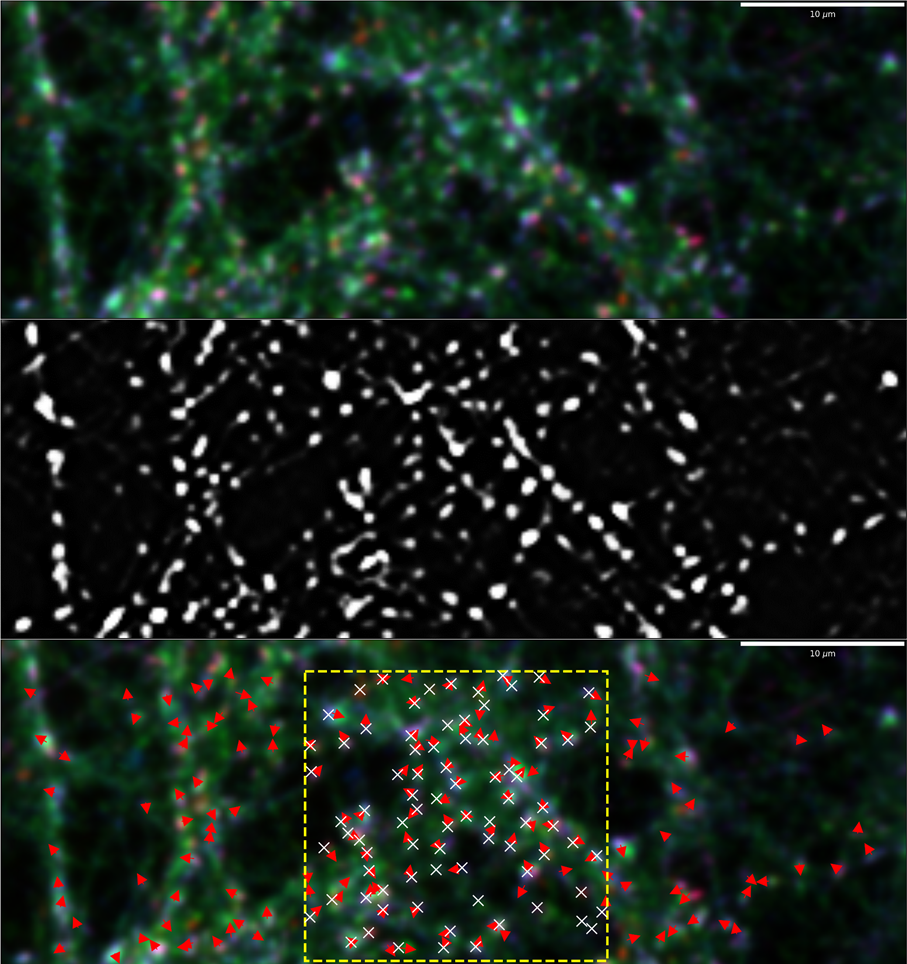

Supplement: S1 Fig — The top image is the original one, the middle is the probability map produced by DoGNet and on the bottom is the overlay of detected synapses on the original image. Detected synapses are denoted with a red arrow, indicating their orientation concerning pre- and postsynaptic sides. The ground truth synapses locations are depicted using white crosses. Yellow bounding box highlights the densely annotated region. (TIF) [file pcbi.1007012.s002.tif]

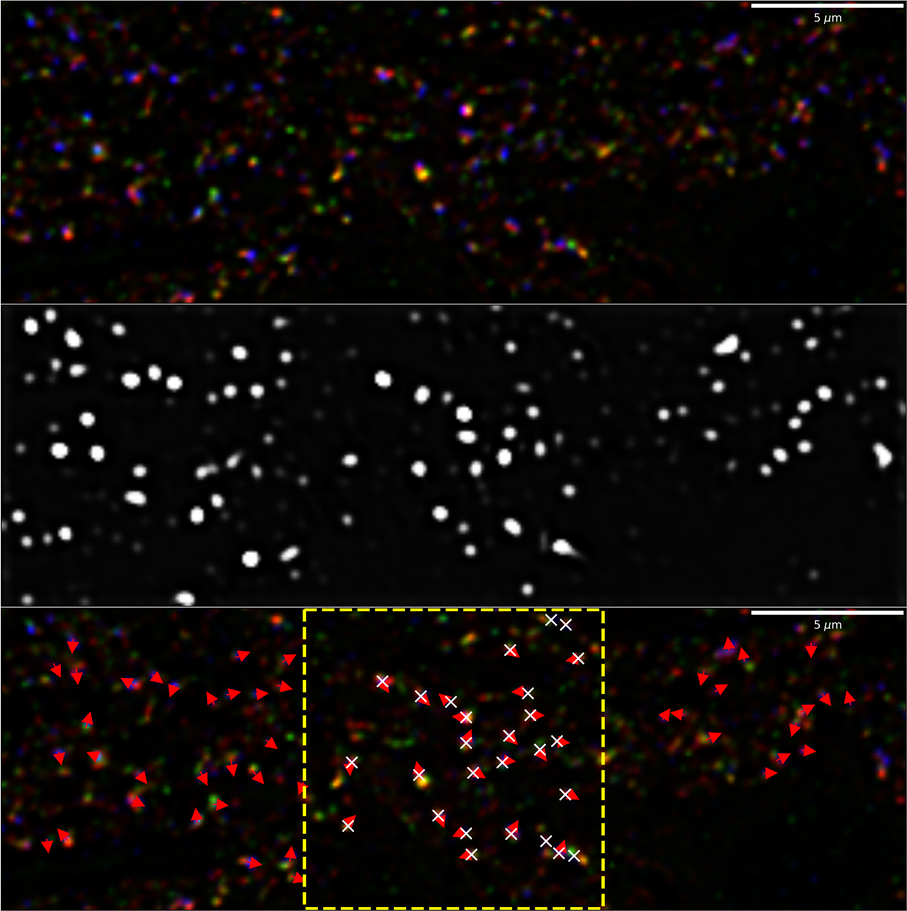

Supplement: S2 Fig — (TIF) [file pcbi.1007012.s003.tif]

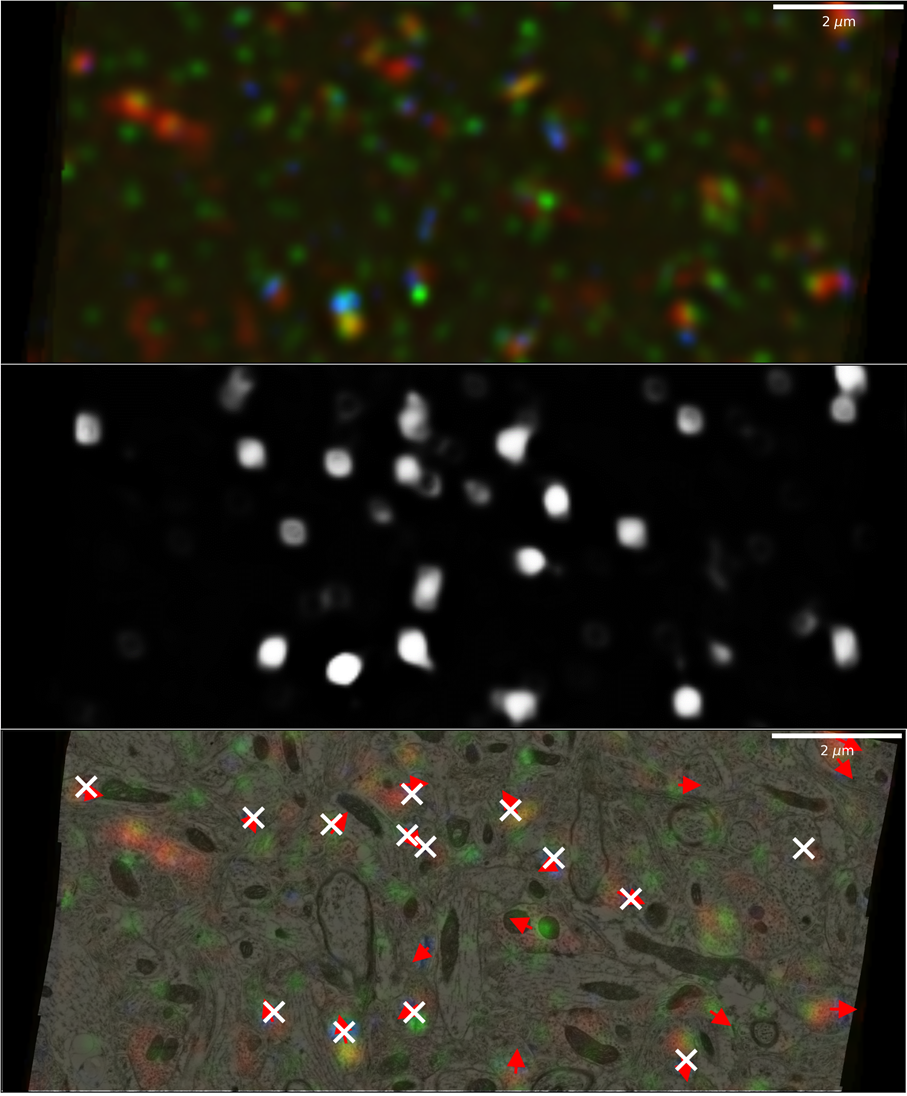

Supplement: S3 Fig — (TIF) [file pcbi.1007012.s004.tif]
